# Birth Weight Reference Percentiles for Chinese

**DOI:** 10.1371/journal.pone.0104779

**Published:** 2014-08-15

**Authors:** Li Dai, Changfei Deng, Yanhua Li, Jun Zhu, Yi Mu, Ying Deng, Meng Mao, Yanping Wang, Qi Li, Shuangge Ma, Xiaomei Ma, Yawei Zhang

**Affiliations:** 1 National Center for Birth Defects Monitoring, West China Second University Hospital of Sichuan University, Chengdu, People's Republic of China; 2 Key Laboratory of Obstetrics & Gynecology and Pediatric Diseases and Birth Defects of the Ministry of Education, West China Second University Hospital of Sichuan University, Chengdu, People's Republic of China; 3 Obstetric and Gynecologic Department, West China Second University Hospital of Sichuan University, Chengdu, People's Republic of China; 4 Yale School of Public Health, Yale University, New Haven, Connecticut, United States of America; Emory University School of Medicine, United States of America

## Abstract

**Objective:**

To develop a reference of population-based gestational age-specific birth weight percentiles for contemporary Chinese.

**Methods:**

Birth weight data was collected by the China National Population-based Birth Defects Surveillance System. A total of 1,105,214 live singleton births aged ≥28 weeks of gestation without birth defects during 2006–2010 were included. The lambda-mu-sigma method was utilized to generate percentiles and curves.

**Results:**

Gestational age-specific birth weight percentiles for male and female infants were constructed separately. Significant differences were observed between the current reference and other references developed for Chinese or non-Chinese infants.

**Conclusion:**

There have been moderate increases in birth weight percentiles for Chinese infants of both sexes and most gestational ages since 1980s, suggesting the importance of utilizing an updated national reference for both clinical and research purposes.

## Introduction

Birth weight for gestational age is a commonly assessed perinatal outcome. Small for gestational age (SGA) is defined as weighing less than the 10^th^ percentile of birth weight and is an important indicator of intrauterine fetal growth restriction (IUGR) [1], [2]. Perinatal and infant morbidity and mortality as well as future adult chronic diseases have been linked to SGA [3], [4], therefore it is important to identify SGA in both clinical and research settings.

Since gestational age-specific birth weight varies among racial groups [5]–[8], nation-specific birth weight references have been developed for several countries [1], [9]–[13]. Although the population in China accounts for one fifth of the world population and each year approximately 16 million babies are born in China [14], no national population-based reference of birth weight currently exists. We used data from the largest National Population-Based Birth Defects Surveillance System (NPBDSS) [14] to construct a national reference of gestational age-specific birth weight percentiles for Chinese born between 2006–2010.

## Methods and Materials

The NPBDSS was established in 2006, and data collected by the NPBDSS have been included in the official system of the National Bureau of Statistics of China since 2007 [14]. This surveillance system covers 64 counties and districts in thirty provinces, municipalities or municipal districts that fall under the central government. This database represents a wide array of geographical locations and socioeconomic status. Details on data collection and quality control of the NPBDSS were described elsewhere [14]. In brief, fetus and neonates of 28 gestational weeks or more born to women living in the surveillance areas for at least one year were recruited and followed. The time period for identifying birth defects was from 28 gestation weeks to 42 days after birth, during which major birth defects (i.e., external malformations and chromosomal aberrations coded according to the International Classification of Diseases 10^th^ edition) diagnosed for the first time were required to be reported. Surveillance staffs at the community, township, or village levels were responsible for birth information collection, verification, and follow-up. By comparing the data with related data from other systems like Birth Certification, Perinatal Death Registry, etc., the information on reported cases or births are checked for accuracy and completeness. In addition, annual surveys are conducted to identify and correct errors and inaccuracies in the collected data. It is required that the under-reporting rate of live births or malformations should be no more than 1% and errors or missing values on the report form should also be no more than 1%.

The gestational age at delivery was calculated in completed weeks from the first day of the last menstrual period (LMP). In the surveillance areas, women with suspected pregnancy have an ultrasound examination for confirmation according to obstetric clinical guidelines. For women with irregular menses and/or bleeding during pregnancy as well as those who could not remember the LMP, gestational ages were estimated based on their ultrasound examination. Birth weight of each neonate was measured by a trained midwife within one hour after birth, recorded to the nearest 5 g, and included in hospital delivery records. The data were then abstracted by trained surveillance staff and entered into a web-based reporting system [14].

**Figure 1:**
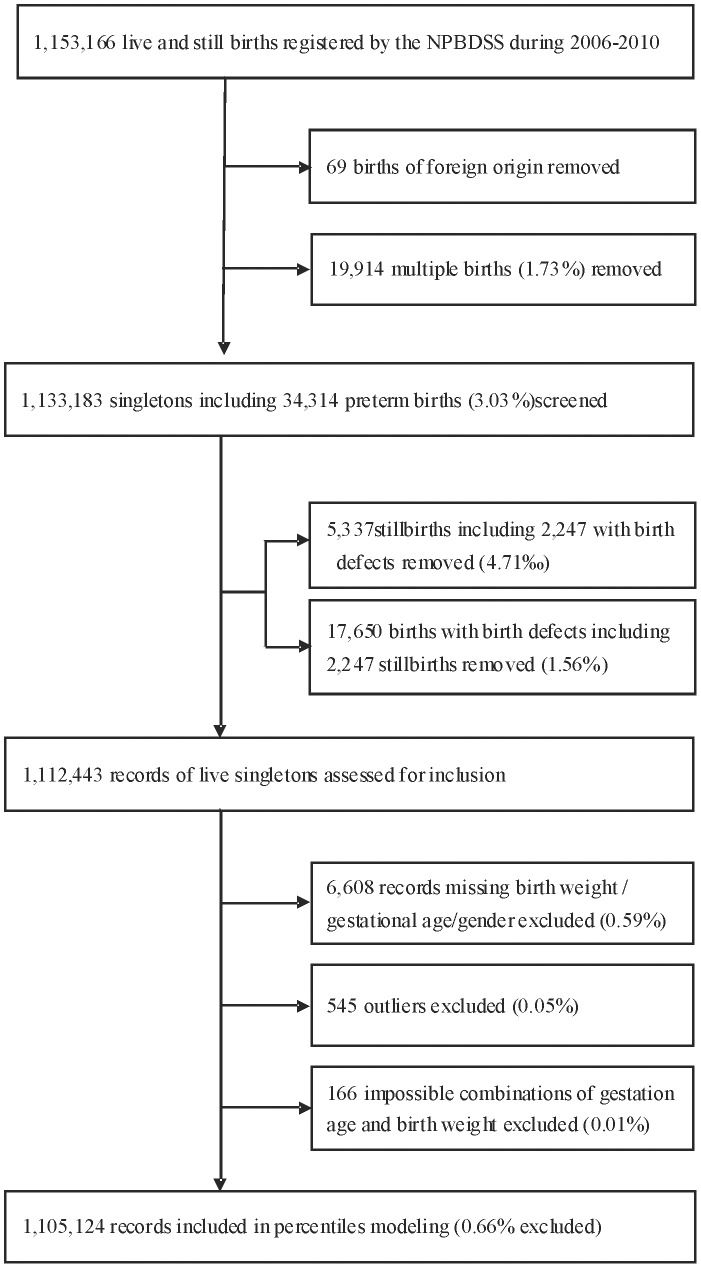
Flow diagram of records selection process.

From October 2006 through September 2010, a total of 1,153,166 live and still births whose gestation age were equal to or greater than 28 weeks were identified by the NPBDSS. Stillbirth was defined as the delivery of a fetus that has died before birth for which there is no possibility of resuscitation. Figure 1 illustrates the records selection process for current study. Stillbirths (n = 5,337, 4.71‰), infants of foreign origin (n = 69), infants from multiple births (n = 19,914, 1.73%), and infants affected by congenital anomalies (n = 17,650, 1.56%), were first excluded from the analysis. Among the rest of 1,112,443 records, 6,608 (0.51%) with missing gestational age or birth weight or gender, and 545 outliers (0.05%) according to previous inclusion criterion [1], were subsequently removed. Finally the procedure proposed by Alexander et al. [1] was adopted to screen records with implausible combinations of gestational age and birth weight. Specifically, gestational age distributions were examined for each 125 g interval of birth weight for preterm infants aged 28–32 weeks. Gestational age values of +/−2.5 standard deviations from the mean were used as cutoffs for implausible records. Under a normal distribution, the cutoffs roughly correspond to the 1^st^ and 99^th^ percentiles. In Alexander et al. [1], manual adjustments of the gestational age “by a week or more” were conducted for certain birth weight intervals. We did not perform such adjustments, due to the infrequent occurrence of abnormal observations. Following this procedure, a total of 7,319 newborns (0.66%) were removed from downstream analysis, yielding a final sample size of 1,105,214 for this study.

For statistical analysis, we first conducted a linear regression analysis and investigated maternal and infant characteristics that might affect birth weight. Since fitting smooth curves on sample quantiles of segmented age groups may demand a large sample size and lose information from nearby groups, we utilized the lambda-mu-sigma (LMS) method for the primary analysis of birth weight for specific gestational ages. The LMS method, which has been used in multiple reference curve studies, adopts a Box-Cox transformation based semiparametric technique and solves penalized likelihood equations. The centiles can be briefly summarized by the *L* (Box-Cox power), *M* (median) and *S* (coefficient variation), which are natural cubic splines with knots at each *T_j_* (gestation week) as described in Cole and Green's paper [15]. The aforementioned analysis was achieved using R package VGAM [16]. To evaluate the impact of employing previous percentiles for the current study cohort, we calculated the relative percentual differences for the 10^th^, 50^th^ and 90^th^ percentiles between our data and those from other references as:

Relative percentual difference  =  (Other_perc_ - China_perc_)/China_perc_×100. Here, the China_perc_ represents the percentiles calculated from our study, while Other_perc_ denotes the percentiles published previously.

## Results

This study included 53.4% male and 46.6% female births. Urban and rural births accounted for 46.4% and 53.6% of the cohort, respectively, while newborns whose mothers lived in coastal regions, inland, and remote areas accounted for 44.0%, 29.9%, and 26.0% of all births respectively. The vast majority (93.2%) of the mothers were Han Chinese, and the rest (6.8%) were minorities. Most (74.0%) mothers aged 20–29 years at the time of delivery, with few (1.3%) aged ≤20 years and 6.7% aged ≥35 years. More than 70% of infants were born to primiparous women (73.0% for boys and 77.0% for girls). (Table 1) Both maternal (age, ethnicity, parity, and residence location/birth area) and infant (gestational age and gender) characteristics were associated with birth weight (Table S1).

**Table 1:** Characteristics of the study population.

Groups	Boys		Girls		Total	
	n	%	n	%	n	%
Birth area						
Urban	270428	45.83	242612	47.10	513040	46.42
Rural	319596	54.17	272488	52.90	592084	53.58
Geographic region						
Coastal	258534	43.82	227807	44.23	486341	44.01
Inland	178843	30.31	152087	29.53	330930	29.95
Remote	152647	25.87	135206	26.25	287853	26.05
Maternal age (years)*						
<20	7808	1.32	7061	1.37	14869	1.35
20–24	186177	31.59	169338	32.91	355515	32.21
25–29	247321	41.96	215344	41.85	462665	41.91
30–34	108333	18.38	89006	17.30	197339	17.88
35–39	34396	5.84	29154	5.67	63550	5.76
≥40	5364	0.91	4603	0.89	9967	0.90
Maternal ethnicity						
Han	549866	93.19	480552	93.29	1030418	93.24
Minority	40158	6.81	34548	6.71	74706	6.76
Parity#						
1	430681	73.01	396459	76.99	827140	74.87
2	148772	25.22	112149	21.78	260921	23.62
≥3	10412	1.77	6336	1.23	16748	1.52

*1219 births with unknown maternal age.

# 315 births with unknown parity.

**Figure 2:**
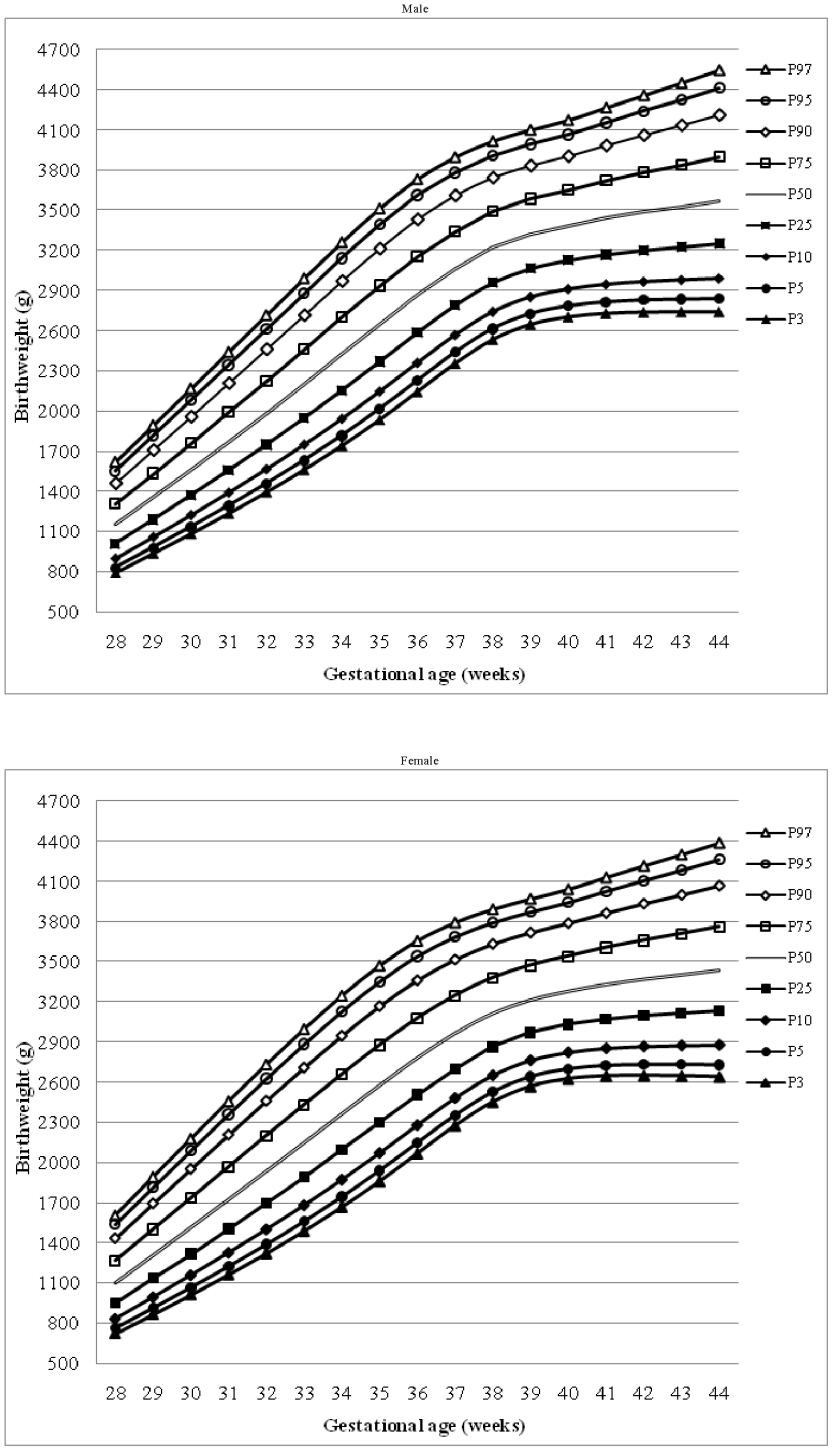
Percentile charts for Chinese newborns. P3, P5 to P97 denote the 3^rd^, 5^th^ to 97^th^ percentile curves, respectively.

**Figure 3:**
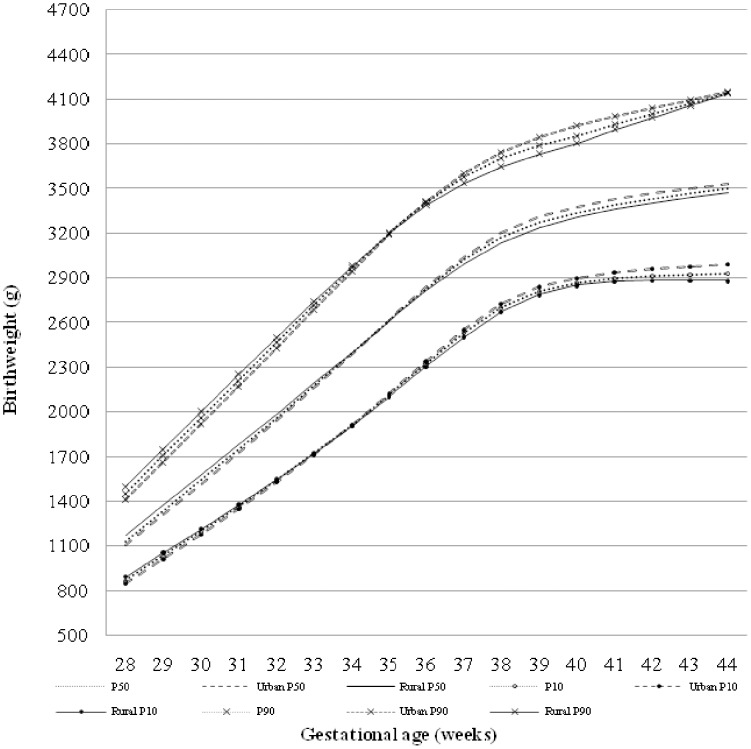
Urban-rural variations of the 10^th^, 50^th^ and 90^th^ birth weight percentiles for Chinese infants irrespective of gender.

Based on the smooth-estimated percentile values (Table 2), reference charts for male and female newborns were generated (Figure 2). As expected, the corrected median birthweights for boys at 28–44 weeks were 2.0–4.5% heavier than for girls. Notably, greater gender differences were observed in the 3^rd^, 5^th^, 10^th^, 25^th^ and 50^th^ birthweight percentiles for preterm births (Figure 2), while for term births, male predominance in birth weight was found in all percentiles (Table 2). Significant urban-rural variations of smoothed birthweight percentiles were also identified (Figure 3). In brief, percentiles for urban term infants were larger than those for rural term births, but larger percentiles were found for rural early preterm babies (particularly ≤32 weeks of gestation). We constructed the smooth-estimated percentile values for Han infants only (Table S2), which showed no significant differences as compared to the percentiles based on all infants.

**Table 2:** Smoothed birth weight percentiles for Chinese newborns during 2006–2010.

Gestation (weeks)	Male	Female
	Number	Mean (SD)	P3	P5	P10	P25	P50	P75	P90	P95	P97	Number	Mean (SD)	P3	P5	P10	P25	P50	P75	P90	P95	P97
28	168	1252(198)	789	830	895	1011	1152	1307	1458	1553	1618	129	1166(210)	721	763	830	952	1102	1268	1432	1537	1607
29	166	1374(224)	934	981	1056	1191	1355	1533	1707	1818	1892	123	1336(225)	864	913	992	1135	1309	1502	1692	1813	1895
30	251	1584(267)	1081	1134	1220	1373	1558	1760	1957	2082	2166	192	1525(276)	1011	1067	1157	1319	1517	1735	1950	2087	2180
31	366	1767(317)	1233	1293	1388	1559	1765	1990	2208	2346	2439	265	1736(349)	1162	1224	1325	1506	1726	1968	2206	2358	2460
32	693	1939(321)	1392	1457	1563	1750	1977	2223	2462	2613	2714	465	1920(359)	1320	1389	1499	1697	1937	2201	2459	2623	2734
33	837	2120(316)	1560	1631	1746	1949	2194	2460	2717	2880	2989	574	2068(332)	1487	1561	1680	1893	2150	2432	2707	2881	2998
34	1682	2359(385)	1740	1816	1939	2156	2417	2699	2972	3144	3259	1232	2293(408)	1667	1745	1871	2095	2365	2659	2945	3126	3248
35	3134	2591(405)	1934	2015	2143	2370	2642	2934	3215	3393	3511	2453	2535(417)	1860	1942	2071	2302	2578	2877	3167	3349	3472
36	8602	2852(459)	2143	2225	2356	2586	2860	3153	3434	3611	3729	6574	2784(461)	2067	2149	2279	2509	2783	3077	3360	3538	3657
37	31949	3078(407)	2355	2437	2565	2791	3058	3342	3613	3782	3895	24400	2989(404)	2276	2356	2482	2704	2967	3247	3515	3683	3794
38	101207	3244(392)	2534	2613	2739	2958	3215	3488	3746	3907	4014	82036	3140(381)	2453	2530	2652	2865	3115	3380	3632	3789	3893
39	187452	3336(387)	2647	2725	2849	3065	3317	3584	3836	3993	4098	163122	3234(374)	2569	2645	2764	2972	3216	3473	3717	3869	3970
40	205506	3390(390)	2704	2783	2908	3127	3382	3652	3907	4065	4171	183982	3291(376)	2627	2703	2824	3035	3281	3541	3787	3940	4042
41	40065	3473(416)	2730	2812	2943	3170	3437	3719	3987	4153	4264	41272	3360(402)	2648	2728	2854	3074	3332	3605	3863	4024	4131
42	6839	3461(430)	2739	2826	2963	3202	3483	3781	4064	4240	4357	7219	3358(416)	2652	2735	2868	3099	3371	3659	3933	4104	4217
43	943	3447(444)	2742	2832	2976	3228	3524	3839	4138	4325	4450	914	3307(421)	2648	2736	2875	3118	3404	3709	3999	4181	4301
44	164	3462(489)	2741	2836	2987	3252	3564	3897	4215	4413	4545	148	3368(476)	2642	2733	2879	3135	3437	3759	4067	4260	4388

Mean (SD) represent the observed mean birth weights for gestational age and corresponding standard deviations. P3, P5 to P97 denote smoothed values for the 3^rd^ and corresponding percentiles.

**Figure 4:**
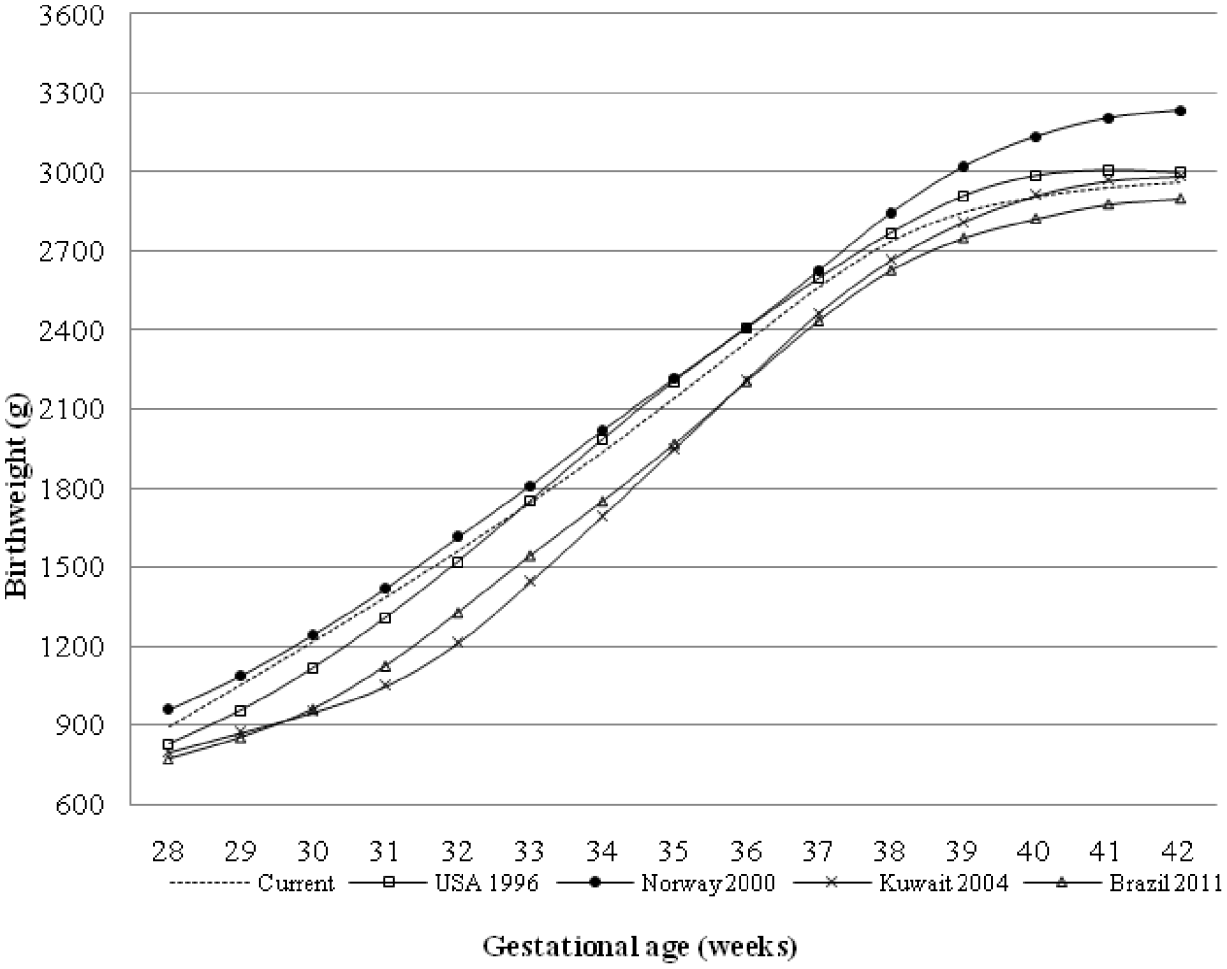
Comparison of the 10^th^ birth weight percentiles for boys between the current study, USA, Norway, Kuwait and Brazil.

Using the values from the current study cohort as references, Table 3 showed relative differences for the 10^th^ and 50^th^ percentiles compared to previously published charts [1], [9]–[13], [17]–[20]. The general characteristics of these studies are presented in Table 4. Negative numbers are shown when the current percentiles are larger than the previous ones, suggesting that relative birth weight will likely be overestimated if older percentile references are used for the current population. On the other hand, positive numbers will likely result in underestimation if other references are used. For example, the SGA would be overestimated for the majority of newborns in our current study population except for very preterm infants (infants ≤30 gestational weeks) if the China 1992 references [20] are used (Table 3). The degree of overestimation or underestimation from using previously published references could be as great as 24.5% for SGA and 14.4% for medium birth weight. Greater differences of the 10^th^ percentiles were found at almost all gestation weeks between several national references. As illustrated in Figure 4, the values of 10^th^ percentiles at each gestation week in the current study were higher than those of Brazilian boys but lower than those of Norwegian male infants.

**Table 3:** Relative percentual differences in the 10^th^ and 50^th^ percentiles of birth weight between the current reference and previously published references.

Gestation (weeks)	China 1992 [20]		USA 1995* [17]		USA 1996 [1]		Australia 1999 [18]		Australia 2012 [9]		Norway 2000# [11]		Canada 2001 [10]		Kuwait 2004 [19]		Scotland 2008* [13]		Brazil 2011 [12]	
	Male	Female	Male	Female	Male	Female	Male	Female	Male	Female	Male	Female	Male	Female	Male	Female	Male	Female	Male	Female
**P10**																				
28	19.11	7.59	−7.26	7.59	−7.49	−3.86	−5.03	−8.43	−5.70	−7.95	7.26	2.41	−4.69	−3.37	−10.95	−11.57	−6.93	−9.88	−13.97	−11.45
29	5.59	1.61	−7.67	1.61	−9.47	−6.75	−10.04	−10.28	−8.71	−12.30	3.22	0.30	−8.71	−8.97	−17.42	−15.12	−9.19	−12.30	−19.32	−17.74
30	−4.26	−2.51	−6.15	−2.51	−8.44	−6.22	−11.48	−9.68	−10.57	−10.98	2.05	0.26	−9.92	−11.67	−22.21	−20.74	−10.16	−11.58	−21.31	−20.66
31	−8.50	−5.21	−3.46	−5.21	−5.76	−3.55	−5.62	−13.96	−8.50	−10.19	2.31	0.75	−9.29	−11.85	−24.50	−24.45	−10.01	−11.40	−19.02	−18.49
32	−9.60	−6.74	−0.83	−6.74	−2.69	−0.27	−10.43	−10.61	−8.51	−10.07	3.33	1.73	−7.61	−10.21	−22.46	−23.02	−10.43	−11.21	−15.10	−14.54
33	−9.22	−7.56	1.66	−7.56	0.29	2.68	−6.07	−9.52	−6.19	−7.14	3.67	2.98	−5.61	−7.86	−17.35	−19.11	−9.91	−10.42	−11.63	1.01
34	−8.25	−7.96	3.66	−7.96	2.37	4.22	−5.11	−5.93	−4.07	−5.72	4.18	3.96	−3.76	−5.51	−12.58	−15.82	−9.23	−8.93	−9.75	−9.14
35	−7.47	−8.21	4.53	−8.21	2.89	4.25	−1.54	−1.98	−2.94	−4.39	3.36	3.57	−2.43	−3.52	−9.10	−12.55	−8.49	−7.44	−8.26	−7.77
36	−7.09	−8.38	4.63	−8.38	2.16	3.29	−1.53	−2.59	−2.59	−3.55	2.29	2.24	−1.49	−2.28	−6.15	−8.78	−6.88	−6.76	−6.41	−5.22
37	−6.98	−8.38	4.29	−8.38	1.21	2.38	−0.58	−2.10	−0.97	−2.10	2.34	1.93	−0.51	−1.21	−3.90	−5.80	−4.41	−5.36	−5.03	−4.88
38	−6.28	−7.47	4.24	−7.47	1.10	2.34	1.50	0.30	2.23	1.43	3.87	2.94	0.99	0.23	−2.67	−4.00	−0.66	−1.92	−4.05	−4.03
39	−4.49	−5.39	5.30	−5.39	2.07	3.18	3.19	2.03	3.55	2.39	6.00	4.74	3.26	2.21	−1.37	−2.50	2.35	1.34	−3.47	−3.76
40	−2.34	−2.73	6.60	−2.73	2.68	3.72	5.57	4.46	6.26	5.35	7.81	6.76	5.88	4.64	0.07	−0.85	6.12	4.50	−2.92	−3.40
41	−0.95	−0.63	6.52	−0.63	2.17	3.29	8.05	6.87	9.41	8.27	8.90	7.74	8.02	6.90	0.82	0.32	9.45	7.64	−2.21	−2.59
42	−0.91	−0.07	4.62	−0.07	1.18	2.34	8.34	7.39	9.69	8.44	9.18	7.74	9.11	8.58	0.71	0.56	10.43	8.40	−2.13	−2.37
**P50**																				
28	7.12	0.09	8.94	6.62	3.82	8.53	1.56	−2.90	1.13	−1.09	6.77	3.45	1.82	0.64	−2.26	−5.81	−0.43	−2.72	1.22	0.45
29	1.33	−2.75	8.49	5.81	2.88	6.49	−1.11	−4.51	−3.25	−5.27	3.32	1.22	−1.70	−3.82	−6.72	−10.08	−3.47	−6.65	−5.31	−4.97
30	−2.05	−4.81	8.79	6.46	5.07	7.91	−4.36	−6.39	−3.85	−7.71	2.05	0.86	−3.27	−5.93	−8.60	−13.32	−4.81	−7.32	6.10	6.26
31	−4.02	−6.20	9.35	7.76	8.67	11.12	−5.38	−7.88	−4.82	−7.88	2.55	1.39	−3.80	−6.55	−9.35	−14.37	−5.04	−8.46	−3.85	−4.06
32	−5.26	−7.12	9.76	8.67	11.43	13.73	−4.40	−8.11	−4.91	−8.11	3.69	2.22	−3.59	−6.20	−9.76	−12.49	−5.97	−9.19	0.25	0.21
33	−6.06	−7.67	9.62	9.07	12.03	14.33	−3.37	−5.12	−4.01	−6.47	4.38	3.02	−3.05	−5.35	−9.07	−9.58	−5.88	−9.02	3.24	3.35
34	−6.70	−7.95	9.23	8.46	10.34	12.77	−2.77	−4.44	−3.19	−5.29	5.09	3.81	−2.36	−4.19	−7.20	−7.65	−5.50	−7.48	3.43	3.59
35	−7.23	−8.07	8.25	7.45	7.15	9.81	−1.97	−3.41	−2.42	−3.80	5.41	4.34	−1.59	−2.79	−4.81	−6.13	−4.50	−5.28	2.12	2.29
36	−7.59	−7.98	7.17	6.36	3.99	6.86	−1.40	−2.26	−1.40	−2.62	5.59	4.74	−0.52	−1.40	−2.69	−4.13	−2.38	−3.45	0.98	1.19
37	−7.52	−7.45	6.44	5.49	1.93	5.06	0.72	−0.24	0.72	−0.07	5.79	5.16	0.72	0.03	−1.50	−2.26	0.16	−1.18	0.07	0.30
38	−6.72	−6.32	6.22	5.30	1.49	4.75	2.95	1.77	3.58	2.73	6.69	6.10	2.33	1.73	−0.84	−1.28	3.05	1.96	−0.40	−0.32
39	−5.03	−4.60	7.02	5.88	2.50	5.72	4.31	3.23	4.61	3.86	8.38	7.43	4.46	3.67	0.21	−0.65	4.91	4.23	−0.51	−0.81
40	−3.22	−2.90	8.07	6.52	3.34	6.52	6.45	5.15	7.04	6.07	10.14	8.96	6.83	5.76	1.21	0.21	7.89	6.61	−0.38	−1.01
41	−2.21	−1.98	8.23	6.69	2.62	5.85	8.52	6.84	9.25	8.19	10.85	9.69	8.61	7.32	0.93	0.69	10.36	8.79	−0.41	−1.20
42	−2.24	−2.34	7.52	5.90	1.12	4.48	8.53	7.09	9.68	8.28	10.54	9.46	9.53	8.42	−0.34	0.53	10.71	8.96	−0.95	−1.81

Negative numbers indicate the current percentiles are larger than the previous ones, while positive numbers suggest current ones are smaller.

*References for infants born to multiparous women.

# Presented mean values because the 50^th^ percentiles by gender are not available. Numbers in brackets represent the references in text.

**Table 4:** Selected published gestational-age-specific birth weight centiles for comparison.

Country	Sample size	Population-based	Years	Method of assessing GA
China, current	1,105,124	Yes	2006–2010	LMP
China 1992 [20]	24,150	No	1986–1987	LMP
USA 1995 [17]	3,427,009	Yes	1989	LMP
USA 1996 [1]	3,134,879	Yes	1991	LMP
Australia 1999 [18]	761,902	Yes	1991–1994	LMP+ clinical estimate
Australia 2012 [9]	2,528,641	Yes	1998–2007	LMP+ ultrasound
Norway 2000 [11]	1,800,000	Yes	1967–1998	LMP
Canada 2001 [10]	676,605	Yes	1994–1996	Ultrasound
Kuwait 2004 [19]	35,768	No	1998–2000	Ultrasound
Scotland 2008 [13]	100,133	Yes	1998–2003	LMP+ ultrasound
Brazil 2011 [12]	7,993,166	Yes	2003–2005	LMP

Numbers in brackets represent the references in text. GA is the abbreviation of gestational age. LMP represents the method that was used to calculate GA based on the last menstrual period.

## Discussion

This study represents the first national population-based, gestational age-specific reference of birth weight for Chinese singleton newborns based on a large and nationally representative database. The Chinese Ministry of Health developed a reference of birth weight for gestational age in 1975 based on data from a survey conducted in nine cities, and the reference has been updated every ten years since then [21]. However, this reference was only for term births. In the mid 1980s, the Chinese Ministry of Health conducted another cross-sectional survey in fifteen cities involving 24,150 live singleton births and developed gestational age-specific reference for birth weight in 1992 [20]. Although this reference included preterm births, the study population was not a nationally representative sample. In addition, during the past two decades there have been considerable changes in both maternal and infant characteristics, such as an increase in maternal age at delivery, improved education level of the mothers, increases in infant weight and length, as well as improvements in prenatal nutritional status [21], [22]. Older references may be obsolete in evaluating contemporary Chinese newborns.

This newly developed reference shows that medium birth weight and 10^th^ percentiles are larger for term and moderate preterm births but are smaller for very preterm births compared to the 1992 Chinese reference [20]. This phenomenon could be due to improved prenatal care, such as advances in neonatal intensive care during the past two decades, which has improved the survival of very preterm births with very low birth weight. In addition, improved nutritional status during the past two decades may have contributed to a heavier birth weight for term births.

When compared with the references from developed countries such as the United States [1], Australia [9], and Canada [10], the current Chinese median birth weights for gestational age were smaller particularly for term and moderate preterm newborns. Notably, the current 10^th^ percentiles were smaller than those for Norwegians at all gestation weeks, but larger than such percentiles for Brazilians. Although mechanisms underlying the racial differences in birth weight patterns remain unclear, previous studies have suggested that environmental factors may be more important than genetic backgrounds [23], [24]. It has also been suggested that racial disparities are more evident for birth weight than for other neonatal growth parameters [5], [25]–[27].

Socioeconomic status [24], [28], [29] and other maternal characteristics [30], [31] have been associated with birth weight. Consistent with earlier studies [13], [24], [28]–[33], the current study found that urban term infants who generally had better socioeconomic conditions had higher birth weights than rural term infants. The inverse urban-rural pattern in percentiles of very preterm babies strongly indicates the effects of environmental factors on birth weight. When compared to rural regions, better nutritional status and prenatal health care in urban areas may contribute to a higher live birth rate for fetuses prone to be premature and heavier birth weight for term babies. In our study, older women tended to give birth to heavier babies than younger women, and term newborns with a higher birth order weighed more than firstborns. Other factors such as delivery type (cesarean section) may affect birth weight distributions, but the effects on percentiles can't be assessed due to limits of data.

As noted previously, SGA is an important indicator of fetal growth restriction, and since there is a high rate of false-positive and false-negative diagnosis of IUGR, a customized chart of birth weight percentiles has been recommended [2]. However, evidence that customized birth weight percentiles are a better predictor of IUGR than population-based gestational-age specific birth weight percentiles is inconsistent [34]–[36]. Furthermore, although fetal weight estimation using the customized birth weight percentiles has led to more accurate predictions of adverse perinatal outcomes [2], fetal weights are not routinely assessed in clinical practice in China. Therefore, population-based birth weight percentiles for gestational age have important implications in both clinical and research settings.

A major strength of the current study is the quality of data, which were obtained from the NPBDSS, a large and well-documented national registry designed to represent populations from a large number of geographic locations. Distributions of ethnic and urban-rural groups in the current study are highly comparable to those from the National Census 2010 (http://www.stats.gov.cn/tjgb/rkpcgb/). In our study, 6.8% of newborns were minorities, similar to the percentage observed in Census 2010 (8.5%). Urban births accounted for 46.4% of our overall study population, and the proportion from Census 2010 was 49.7%. Studies of birth registry data have the potential for error in the estimation of gestational age, measurement of birth weight, and data transcription. To reduce the possibility of error in our study, 0.66% of the records were removed from final analysis due to missing key variables or outlier values. Although variations in birth weight data collected at our various sites could influence the accuracy of percentiles, discrepancies in measurement were likely minimal due to the high quality of prenatal care and professionally trained midwives.

In summary, our novel gestational age-specific birth weight percentiles for contemporary Chinese singleton births are based on data from the largest national registry, making this version a more accurate and relevant resource for clinical practice, public health research, and health policy. It represents the first national reference for clinicians and researchers and may promote the recognition of SGA as a different concept from low birth weight. Although both conditions are associated with poor health outcomes and a higher incidence of future diseases such as diabetes, heart disease and even cognitive disabilities [4], [37]–[40], identification of SGA in fetuses may provide an opportunity for early intervention.

## Supporting Information

Table S1 **Linear regression analysis on the relationship between birth weight and maternal/infant characteristics.**
(DOCX)

Table S2 **Smoothed birth weight percentiles for Han Chinese infants by gender during 2006–2010.**
(DOCX)

## References

[1] Alexander GR, Himes JH, Kaufman RB, Mor J, Kogan M (1996) A United States national reference for fetal growth. Obstet Gynecol 87: 163–168. 8559516 10.1016/0029-7844(95)00386-X

[2] Resnik R (2007) One size does not fit all. Am J Obstet Gynecol 197: 221–222. 17826399 10.1016/j.ajog.2007.07.019

[3] Wilcox AJ (2001) On the importance–and the unimportance–of birthweight. Int J Epidemiol 30: 1233–1241. 11821313 10.1093/ije/30.6.1233

[4] Langley-Evans SC, McMullen S (2010) Developmental origins of adult disease. Med Princ Pract 19: 87–98. 20134170 10.1159/000273066

[5] Madan A, Holland S, Humbert JE, Benitz WE (2002) Racial differences in birth weight of term infants in a northern California population. J Perinatol 22: 230–235. 11948387 10.1038/sj.jp.7210703

[6] Thomas P, Peabody J, Turnier V, Clark RH (2000) A new look at intrauterine growth and the impact of race, altitude, and gender. Pediatrics 106: E21. 10920177 10.1542/peds.106.2.e21

[7] Wang X, Guyer B, Paige DM (1994) Differences in gestational age-specific birthweight among Chinese, Japanese and white Americans. Int J Epidemiol 23: 119–128. 8194906 10.1093/ije/23.1.119

[8] Zhang B, Yip R, Wen F, Wang B (1997) Comparison of birth weight by gestational age between China and the United States. Chin Med J (Engl) 110: 148–151. 9594289

[9] Dobbins TA, Sullivan EA, Roberts CL, Simpson JM (2012) Australian national birthweight percentiles by sex and gestational age, 1998–2007. Med J Aust 197: 291–294. 22938128 10.5694/mja11.11331

[10] Kramer MS, Platt RW, Wen SW, Joseph KS, Allen A, et al (2001) A new and improved population-based Canadian reference for birth weight for gestational age. Pediatrics 108: E35. 11483845 10.1542/peds.108.2.e35

[11] Skjaerven R, Gjessing HK, Bakketeig LS (2000) Birthweight by gestational age in Norway. Acta Obstet Gynecol Scand 79: 440–449. 10857867

[12] Pedreira CE, Pinto FA, Pereira SP, Costa ES (2011) Birth weight patterns by gestational age in Brazil. An Acad Bras Cienc 83: 619–625. 21625798 10.1590/s0001-37652011005000008

[13] Bonellie S, Chalmers J, Gray R, Greer I, Jarvis S, et al (2008) Centile charts for birthweight for gestational age for Scottish singleton births. BMC Pregnancy Childbirth 8: 5. 18298810 10.1186/1471-2393-8-5 PMC2268653

[14] Dai L, Zhu J, Liang J, Wang YP, Wang H, et al (2011) Birth defects surveillance in China. World J Pediatr 7: 302–310. 22015723 10.1007/s12519-011-0326-0

[15] Cole TJ, Green PJ (1992) Smoothing reference centile curves: the LMS method and penalized likelihood. Stat Med 11: 1305–1319. 1518992 10.1002/sim.4780111005

[16] Yee TW (2008) The VGAM Package. R News 8: 28–39.

[17] Zhang J, Bowes WA Jr (1995) Birth-weight-for-gestational-age patterns by race, sex, and parity in the United States population. Obstet Gynecol 86: 200–208. 7617350 10.1016/0029-7844(95)00142-e

[18] Roberts CL, Lancaster PA (1999) Australian national birthweight percentiles by gestational age. Med J Aust 170: 114–118. 10065122 10.5694/j.1326-5377.1999.tb127678.x

[19] Alshimmiri MM, Al-Saleh EA, Alsaeid K, Hammoud MS, Al-Harmi JA (2004) Birthweight percentiles by gestational age in Kuwait. Arch Gynecol Obstet 269: 111–116. 14648179 10.1007/s00404-002-0434-0

[20] Zhang BL (1992) The revised report on gestational age-specific birthweights of boys and girls in 15 cities of China. Chin J Prac Pediar 7: 306–307.

[21] Li H, Zhu Z, Zhang D (2007) [A national survey on growth of children under 7 years of age in nine cities of China, 2005]. Zhonghua Er Ke Za Zhi 45: 609–614. 18021536

[22] Zheng X, Pang L, Tellier S, Tan L, Zhang L, et al (2013) The changing patterns of abortion among married women in China, 1984–2005. Eur J Obstet Gynecol Reprod Biol 166: 70–75. 23219292 10.1016/j.ejogrb.2012.09.016

[23] Bakketeig LS (1998) Current growth standards, definitions, diagnosis and classification of fetal growth retardation. Eur J Clin Nutr 52 Suppl 1S1–4. 9511013

[24] Kramer MS (1998) Socioeconomic determinants of intrauterine growth retardation. Eur J Clin Nutr 52 Suppl 1S29–32 discussion S32–23. 9511017

[25] Yip R, Li Z, Chong WH (1991) Race and birth weight: the Chinese example. Pediatrics 87: 688–693. 2020515

[26] Janssen PA, Thiessen P, Klein MC, Whitfield MF, Macnab YC, et al (2007) Standards for the measurement of birth weight, length and head circumference at term in neonates of European, Chinese and South Asian ancestry. Open Med 1: e74–88. 20101298 PMC2802014

[27] Kierans WJ, Joseph KS, Luo ZC, Platt R, Wilkins R, et al (2008) Does one size fit all? The case for ethnic-specific standards of fetal growth. BMC Pregnancy Childbirth 8: 1. 18179721 10.1186/1471-2393-8-1 PMC2266898

[28] Pearl M, Braveman P, Abrams B (2001) The relationship of neighborhood socioeconomic characteristics to birthweight among 5 ethnic groups in California. Am J Public Health 91: 1808–1814. 11684609 10.2105/ajph.91.11.1808 PMC1446884

[29] Teramoto S, Soeda A, Hayashi Y, Urashima M (2006) Physical and socioeconomic predictors of birthweight in Japan. Pediatr Int 48: 274–277. 16732794 10.1111/j.1442-200X.2006.02203.x

[30] da Veiga PV, Wilder RP (2008) Maternal smoking during pregnancy and birthweight: a propensity score matching approach. Matern Child Health J 12: 194–203. 17551817 10.1007/s10995-007-0238-8

[31] Swamy GK, Edwards S, Gelfand A, James SA, Miranda ML (2012) Maternal age, birth order, and race: differential effects on birthweight. J Epidemiol Community Health 66: 136–142. 21081308 10.1136/jech.2009.088567 PMC4052610

[32] Wang J, Ren AG, Ye RW, Zheng JC, Li S, et al (2007) [Study on the third trimester hemoglobin concentrations and the risk of low birth weight and preterm delivery]. Zhonghua Liu Xing Bing Xue Za Zhi 28: 15–18. 17575924

[33] Ogunyemi D, Manigat-Wilson B, Bazargan M, Pan D (2007) Birth weight for gestational age patterns by ethnicity, gender, and parity in an urban population. South Med J 100: 615–616. 10.1097/SMJ.0b013e318048798c 17593580

[34] Hutcheon JA, Zhang X, Cnattingius S, Kramer MS, Platt RW (2008) Customised birthweight percentiles: does adjusting for maternal characteristics matter? BJOG 115: 1397–1404. 18823489 10.1111/j.1471-0528.2008.01870.x

[35] Hutcheon JA, Zhang X, Platt RW, Cnattingius S, Kramer MS (2011) The case against customised birthweight standards. Paediatr Perinat Epidemiol 25: 11–16. 21133965 10.1111/j.1365-3016.2010.01155.x

[36] Larkin JC, Hill LM, Speer PD, Simhan HN (2012) Risk of morbid perinatal outcomes in small-for-gestational-age pregnancies: customized compared with conventional standards of fetal growth. Obstet Gynecol 119: 21–27. 22183207 10.1097/AOG.0b013e31823dc56e

[37] Barker DJ, Hales CN, Fall CH, Osmond C, Phipps K, et al (1993) Type 2 (non-insulin-dependent) diabetes mellitus, hypertension and hyperlipidaemia (syndrome X): relation to reduced fetal growth. Diabetologia 36: 62–67. 8436255 10.1007/BF00399095

[38] Curhan GC, Willett WC, Rimm EB, Spiegelman D, Ascherio AL, et al (1996) Birth weight and adult hypertension, diabetes mellitus, and obesity in US men. Circulation 94: 3246–3250. 8989136 10.1161/01.cir.94.12.3246

[39] McCarton CM, Wallace IF, Divon M, Vaughan HG Jr (1996) Cognitive and neurologic development of the premature, small for gestational age infant through age 6: comparison by birth weight and gestational age. Pediatrics 98: 1167–1178. 8951271

[40] Shenkin SD, Starr JM, Deary IJ (2004) Birth weight and cognitive ability in childhood: a systematic review. Psychol Bull 130: 989–1013. 15535745 10.1037/0033-2909.130.6.989

