# Supplementary material for: Birth Weight Reference Percentiles for Chinese
Source: PLoS One. 2014 Aug 15;9(8):e104779. doi: 10.1371/journal.pone.0104779 (PMC4134219; doi:10.1371/journal.pone.0104779)
Supplement: Table S1 — Linear regression analysis on the relationship between birth weight and maternal/infant characteristics. (DOCX) [file pone.0104779.s001.docx]

**Table S1 Linear regression analysis on the relationship between birth weight and maternal/infant characteristics.**

| Variables | Variable assignment | Coefficient estimates | t Values | P values |
| --- | --- | --- | --- | --- |
| Gestational age | Continuous variable | 113.5 | 389.0 | <10^-16^ |
| Maternal age |  | 10.3 | 109.0 | <10^-16^ |
| Parity (birth order) |  | -23.1 | -26.7 | <10^-16^ |
| Gender | Boy=1, girl=2 | -102.4 | -137.8 | <10^-16^ |
| Maternal ethnicity | Han=1, Other =2 | -71.3 | -48.2 | <10^-16^ |
| Birth area (residence location) | Urban=1, rural=2 | -59.8 | -76.4 | <10^-16^ |

Reference was set to group 1 for each categorical variable.
