# Supplementary material for: Birth Weight Reference Percentiles for Chinese
Source: PLoS One. 2014 Aug 15;9(8):e104779. doi: 10.1371/journal.pone.0104779 (PMC4134219; doi:10.1371/journal.pone.0104779)
Supplement: Table S2 — Smoothed birth weight percentiles for Han Chinese infants by gender during 2006–2010. (DOCX) [file pone.0104779.s002.docx]

**Table S2 Smoothed birth weight percentiles for Han Chinese infants by gender during 2006-2010.**

| Gestation  weeks | Number | Mean±SD | Boys | | | | | | | | |  | Number | Mean±SD | Girls | | | | | | | | |
| --- | --- | --- | --- | --- | --- | --- | --- | --- | --- | --- | --- | --- | --- | --- | --- | --- | --- | --- | --- | --- | --- | --- | --- |
|  |  |  | P3 | P5 | P10 | P25 | P50 | P75 | P90 | P95 | P97 |  |  |  | P3 | P5 | P10 | P25 | P50 | P75 | P90 | P95 | P97 |
| 28 | 156 | 1254(200) | 784 | 825 | 890 | 1007 | 1149 | 1305 | 1457 | 1554 | 1619 |  | 115 | 1157(203) | 721 | 762 | 829 | 949 | 1097 | 1260 | 1422 | 1525 | 1595 |
| 29 | 147 | 1359(227) | 930 | 977 | 1053 | 1188 | 1353 | 1533 | 1708 | 1820 | 1895 |  | 115 | 1334(216) | 865 | 914 | 992 | 1133 | 1305 | 1496 | 1685 | 1805 | 1887 |
| 30 | 232 | 1588(267) | 1079 | 1132 | 1218 | 1372 | 1558 | 1761 | 1959 | 2085 | 2170 |  | 172 | 1526(280) | 1013 | 1068 | 1157 | 1318 | 1515 | 1732 | 1945 | 2082 | 2174 |
| 31 | 339 | 1770(324) | 1232 | 1292 | 1388 | 1559 | 1766 | 1992 | 2211 | 2351 | 2444 |  | 246 | 1736(347) | 1165 | 1227 | 1326 | 1506 | 1725 | 1967 | 2204 | 2355 | 2458 |
| 32 | 638 | 1940(317) | 1393 | 1459 | 1564 | 1752 | 1979 | 2226 | 2466 | 2618 | 2720 |  | 425 | 1915(357) | 1323 | 1391 | 1501 | 1698 | 1938 | 2201 | 2459 | 2624 | 2735 |
| 33 | 791 | 2128(316) | 1562 | 1634 | 1749 | 1953 | 2198 | 2464 | 2722 | 2886 | 2995 |  | 526 | 2066(329) | 1491 | 1565 | 1683 | 1895 | 2153 | 2434 | 2709 | 2884 | 3002 |
| 34 | 1543 | 2360(384) | 1744 | 1821 | 1943 | 2161 | 2422 | 2704 | 2978 | 3150 | 3266 |  | 1120 | 2297(406) | 1672 | 1750 | 1875 | 2099 | 2369 | 2663 | 2950 | 3131 | 3254 |
| 35 | 2910 | 2597(405) | 1940 | 2020 | 2149 | 2376 | 2647 | 2940 | 3222 | 3400 | 3519 |  | 2275 | 2542(418) | 1866 | 1947 | 2077 | 2307 | 2583 | 2883 | 3173 | 3356 | 3479 |
| 36 | 8006 | 2858(459) | 2150 | 2232 | 2363 | 2593 | 2866 | 3160 | 3441 | 3618 | 3736 |  | 6132 | 2788(460) | 2075 | 2156 | 2286 | 2515 | 2789 | 3083 | 3367 | 3545 | 3665 |
| 37 | 29304 | 3086(407) | 2363 | 2444 | 2573 | 2798 | 3065 | 3348 | 3619 | 3788 | 3901 |  | 22380 | 2997(403) | 2284 | 2364 | 2490 | 2711 | 2974 | 3254 | 3521 | 3689 | 3801 |
| 38 | 93798 | 3250(391) | 2542 | 2621 | 2746 | 2965 | 3221 | 3493 | 3751 | 3912 | 4019 |  | 76243 | 3146(380) | 2462 | 2539 | 2660 | 2872 | 3121 | 3386 | 3638 | 3795 | 3899 |
| 39 | 173726 | 3342(386) | 2655 | 2733 | 2857 | 3071 | 3323 | 3589 | 3841 | 3997 | 4101 |  | 151610 | 3240(373) | 2578 | 2653 | 2772 | 2979 | 3222 | 3479 | 3722 | 3873 | 3974 |
| 40 | 193655 | 3395(388) | 2712 | 2791 | 2916 | 3133 | 3388 | 3656 | 3910 | 4068 | 4173 |  | 173193 | 3296(375) | 2635 | 2711 | 2832 | 3041 | 3286 | 3546 | 3791 | 3944 | 4046 |
| 41 | 37351 | 3480(414) | 2738 | 2820 | 2950 | 3177 | 3443 | 3724 | 3991 | 4156 | 4267 |  | 38394 | 3367(401) | 2657 | 2736 | 2862 | 3081 | 3338 | 3610 | 3868 | 4029 | 4135 |
| 42 | 6283 | 3463(430) | 2746 | 2832 | 2969 | 3208 | 3489 | 3786 | 4069 | 4245 | 4362 |  | 6655 | 3363(415) | 2660 | 2744 | 2876 | 3106 | 3377 | 3665 | 3939 | 4109 | 4223 |
| 43 | 844 | 3452(447) | 2747 | 2838 | 2981 | 3233 | 3530 | 3845 | 4145 | 4332 | 4457 |  | 821 | 3311(419) | 2657 | 2744 | 2883 | 3125 | 3412 | 3716 | 4007 | 4188 | 4309 |
| 44 | 143 | 3463(469) | 2745 | 2840 | 2992 | 3257 | 3570 | 3904 | 4223 | 4422 | 4555 |  | 130 | 3393(480) | 2650 | 2742 | 2888 | 3143 | 3445 | 3768 | 4076 | 4269 | 4398 |

Mean (SD) represent the observed mean birth weights for gestational age and corresponding standard deviations. P3, P5 to P97 denote smoothed values for the 3^rd^ and corresponding percentiles.
